# Mechanism of orthotic therapy for the painful cavus foot deformity

**DOI:** 10.1186/1757-1146-7-2

**Published:** 2014-01-23

**Authors:** Bijan Najafi, James S Wrobel, Joshua Burns

**Affiliations:** 1 Interdisciplinary Consortium on Advanced Motion Performance (iCAMP), Southern Arizona Limb Salvage Alliance (SALSA), University of Arizona College of Medicine, Tucson, AZ, USA; 2 Arizona Center on Aging, University of Arizona College of Medicine, Tucson, AZ, USA; 3 Internal Medicine; Metabolism, Endocrinology and Diabetes Division, University of Michigan Medical School, Ann Arbor, MI, USA; 4 The University of Sydney and The Children’s Hospital at Westmead, Sydney, Australia

**Keywords:** Foot pain, Pes cavus, Plantar pressure, Modeling pain relief, Probability distribution of peak pressure, Dynamic plantar loading index

## Abstract

**Background:**

People who have extremely high arched feet or pes cavus often suffer from substantial foot pain. Custom-made foot orthoses (CFO) have been shown to be an effective treatment option, but their specificity is unclear. It is generally thought that one of the primary functions of CFO is redistributing abnormal plantar pressures. This study sought to identify variables associated with pain relief after CFO intervention.

**Methods:**

Plantar pressure data from a randomized controlled trial of 154 participants with painful pes cavus were retrospectively re-analyzed at baseline and three month post CFO intervention. The participants were randomized to a treatment group given CFO or a control group given sham orthoses.

**Results:**

No relationship between change in pressure magnitude and change in symptoms was found in either group. However, redistribution of plantar pressure, measured with the Dynamic Plantar Loading Index, had a significant effect on pain relief (p = 0.001). Our final model predicted 73% of the variance in pain relief from CFO and consisted of initial pain level, BMI, foot alignment, and changes in both Dynamic Plantar Loading Index and pressure–time integral.

**Conclusion:**

Our data suggest that a primary function of effective orthotic therapy with CFO is redistribution of abnormal plantar pressures. Results of this study add to the growing body of literature providing mechanistic support for CFO providing pain relief in painful foot conditions. The proposed model may assist in better designing and assessing orthotic therapy for pain relief in patients suffering painful cavus foot deformity.

**Trial registration:**

Randomized controlled trial: ISRCTN84913516

## Background

Custom-made foot orthoses have been shown to be an effective treatment option for foot pain in a variety of clinical populations [1]. However, the mechanism by which they produce an effect is not well understood.

There are a number of theoretical explanations, including resisting or facilitating motion; plantar pressure reduction; altered muscle activity; and enhanced proprioception [2,3]. Scientific evaluation of these theories has posed many challenges for researchers and overwhelming support for one particular theoretical model is lacking [4]. It is likely, however, that orthoses have different mechanisms for different types of foot pain.

Patients who have extremely high arched feet, or pes cavus, and associated musculoskeletal foot pain are particularly responsive to orthotic therapy [5]. It has been estimated that 60% of people with pes cavus will experience foot pain, such as metatarsalgia, sesamoiditis and plantar heel pain, all of which are thought to be the result of high, localized plantar pressures [5]. Structurally, the cavus foot deformity has reduced ground contact area and is rigid and less shock absorbent, in contrast to the dynamic adaptability of normal and planus (flat) feet [6]. Management of the painful cavus foot has, therefore, been directed toward the reduction of pressure through the application of pressure relieving insoles.

We previously reported a randomized controlled clinical trial into the efficacy of orthoses for painful pes cavus [5,7]. Participants were either assigned to a group that received customized, polypropylene foot orthoses or to a control group consisting of a flat, nonsupportive, latex foam insole. We demonstrated a statistically and clinically significant effect on foot pain and function for the custom-made foot orthoses over the sham. We also showed improvements in quality of life measures and greater reduction in pressure variables in the intervention group. We concluded that custom-made foot orthoses are beneficial for people with painful pes cavus, postulating a link between the improvements in pain and function and reduced plantar pressures.

Subsequently, however, we found no correlation between change in pressure and change in pain with the use of custom-made foot orthoses [7]. While our initial expectations were based on the reduction in plantar pressure being correlated with reduction of pain and subsequent improvement in function, we were not able to show this to be the case [7].

Recently, a new method to evaluate change in plantar pressure has been developed [8,9]. Since absolute pressure reduction is dependent on many factors that vary between patients and trials, (e.g. walk speed, BMI, antalgic gait), redistribution of pressure might be a more sensitive measure. The Dynamic Plantar Loading Index (DPLI) is a method for investigating the redistribution of plantar pressures. The method is based on the probability distribution of peak pressure time series and is quantified using the Regression Factor [8]. The DPLI is a dynamic plantar loading measure estimated from fitting a person’s plantar pressure probability distribution to a Gaussian distribution [8]. In other words, it is a direct measure of what has been thought to be an important mechanism of how custom-made foot orthoses work: by redistributing plantar pressures.

In summary, DPLI is calculated by the least square regression slope between the experimentally observed plantar pressure magnitude probability distribution and a Gaussian distribution with the same mean, standard deviation, and magnitude. The theoretical value of DPLI is confined between −1 to +1 and a value closer to +1 is interpreted as a better agreement between the DPLI and the Gaussian distribution. Healthy participants have demonstrated to have a DPLI = 0.46 during barefoot walking and DPLI = 0.51 ± 0.15 during shod walking while patients with major foot deformities such as Charcot patients have a negative value [9]. After surgical reconstruction, the Charcot patient’s DPLI approximated healthy participants. On the same note, our initial study demonstrated that DPLI in patients with painful pes cavus (Foot Posture Index (FPI) < −2, average = −4.3 ± 2.8) without corrective insoles is 41% lower (DPLI = 0.30 ± 0.16) than individuals with normal foot posture (effect size = 1.40, p < 10^-7^) [8]. Preliminary testing demonstrated this measure to be independent of gait speed, an important confounding parameter for plantar pressure time-series profile [8,9].

The aim of this study was to model the contribution of the DPLI with other possible predictors of cavus foot pain reduction with the use of custom-made foot orthoses.

## Materials and methods

### Participants

The original trial data were obtained from the principal investigator (JB) and the methods were described previously [5]. In brief, after institutional ethics approval and informed consent, 154 participants (56% male; Age = 50 ± 14 years; BMI = 27.8 ± 6.1 kg/m^2^) with painful pes cavus were enrolled on the basis of reaching a threshold Foot Posture Index (FPI) [10] of −2 or less [11]. FPI is a diagnostic tool that evaluates the multi-segmental and multi-planar aspects of the foot using six criteria that together enable the foot to be scored along a continuum of cavus (supinated) to planus (pronated) features [10]. The six components provide a reliable and valid aggregate score ranging from −12 for highly cavus to +12 for highly planus foot alignment [10,12,13].

Eligible participants were men and women 18 years or older, with painful bilateral pes cavus. Patients were excluded if they had rheumatoid arthritis or active foot ulcers, or if they were taking analgesics, or were unable to walk a distance of 20 m independently.

### Interventions

**Figure 1:**
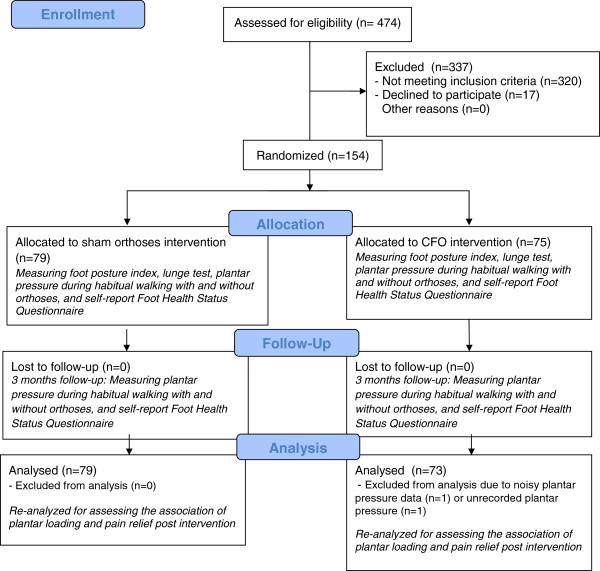
Consort flow diagram.

Participants were randomly assigned to two groups (Figure 1): A control group that was given sham orthoses (3 mm latex foam) and an active treatment group that was prescribed custom-made foot orthoses (standard prescription of a direct milled 3 mm polypropylene) [5]. Both groups were casted with plaster of paris in identical fashion [5]. The methods and criteria of milling for the intervention group has been described in our previous publication [5]. In summary, members of the treatment group were fitted with a pair of custom foot orthoses molded from neutral-suspension plaster casts of the feet by an experienced podiatric physician (JB, the senior author). The casts were scanned using a three dimensional laser scanner, and the orthoses were fabricated from 3 mm polypropylene using a computer aided design–computer-aided manufacturing milling machine to a standardized prescription that had been previously developed and pilot tested [14]. The orthoses were covered with full-length 3 mm Poron Medical urethane (Rogers Corp, Woodstock, Connecticut), which is a commonly used and effective material for absorbing shock and reducing pressure. The key feature of the device is the contoured flexible shell molded to the exact morphological features of the patient’s foot. With the addition of a full-length cushioned top cover, the orthotic device aims to reduce and redistribute abnormal plantar pressures [5].

### Biomechanical assessment of foot orthoses benefit

Biomechanical benefit of custom orthoses was assessed by quantifying the change in dynamic plantar pressure. Plantar pressure assessment was performed using computerized pressure insoles (Pedar®, Novel-Germany) in a standardized shoe (Dunlop Volley; Pacific Dunlop Ltd, Melbourne, Australia) and wearing standardized socks (Brooks; Texas Peak Pty Ltd, Tullamarine, Australia). Participants’ shoe insole was removed and replaced by the randomly allocated orthoses (custom-made or sham) and the pressure insole was applied between the foot and the allocated orthoses i.e. on top of the orthoses. Participants walked a minimum of 40 steps at their self-selected walking speed on a 10 m walkway. The sampling frequency was 50 Hz.

A toolbox was designed and pilot tested in our previous study [8], to characterize and quantify the shape of peak plantar pressure time-series during walking. Three outcomes were extracted using this toolbox including 1) the DPLI 2) the magnitude of 2^nd^ peak pressure (P_M2_) and 3) the relative location of 2^nd^ peak pressure as a percentage of the stance phase (P_Loc2_). Previously, CFO have been shown to influence the ankle moment, thus motivating our attention to the location and amplitude of 2^nd^ peak pressure [15,16].

In our previous study [8], we demonstrated that cavus foot deformity significantly reduces the DPLI on average by 41%, increases the P_Loc2_ by 51%, and decreases the timing to the second peak by 5.8% compared to healthy foot posture. Thus changes in these three parameters together with changes in pressure time integral (PTI) and maximum magnitude of peak pressure were considered as plantar pressure parameters for predicting pain relief after three months of wearing foot orthoses. Furthermore, the alignment of the foot, quantified by Foot Posture Index, and ankle range of motion, quantified by lunge test [17], were both included in the predictive model as well.

**Figure 2:**
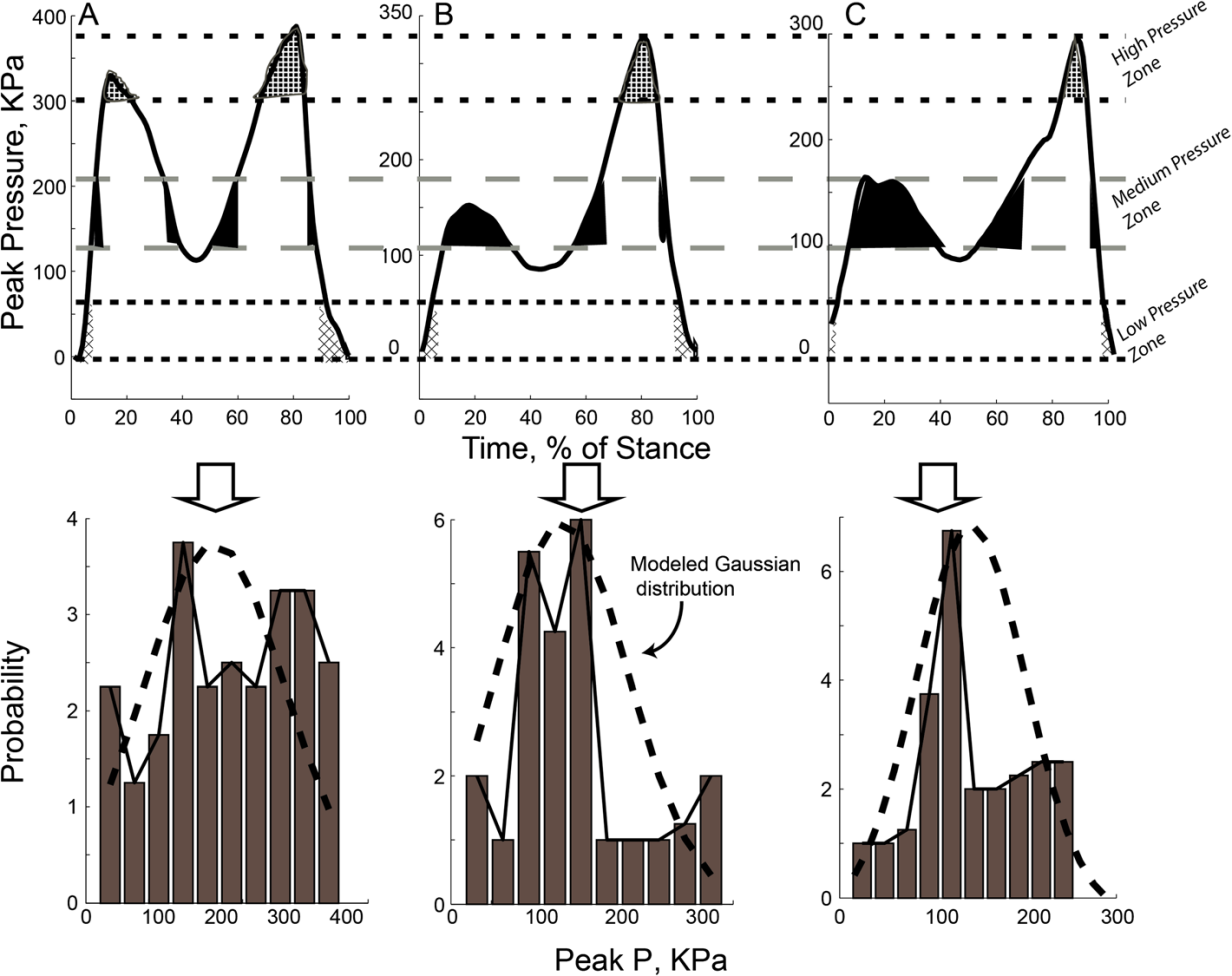
**Plantar pressure magnitude during walking for a typical pes cavus participant not wearing Custom-Made Orthoses, CFO (Figure 2A) and wearing Custom-Made Orthoses (Figure 2B) as well as a typical healthy participant with normal foot alignment (Figure 2C).** In participants with normal foot alignment, there is a greater probability of seeing medium pressure values during stance which make up the central part of the normal distribution

The estimation of the DPLI has been described in our previous publication [8]. In summary, the time-series of peak pressure profile during each stance phase was scaled to 100 samples using a linear interpolation scheme to moderate the effect of gait velocity (stance duration). Then a Gaussian distribution with the same mean, variance, and maximum probability magnitude as the actual data was fitted for each foot and trial using a linear regression model, producing the DPLI [8]. The normal distribution evaluated the probability of observing low, medium or high pressures at any point during stance. For instance, very low and high pressures were expected but usually occur very rapidly (at heel contact and toe off) and therefore account for only a small percentage of the stance phase. These pressures therefore make up the tail ends of the distribution. There was a greater probability of seeing medium pressures during stance and these intermediate pressures made up the central part of the normal distribution (Figure 2). The theoretical value of DPLI is expected to range from - 1 to + 1 and a value close to +1 (regression coefficient =1) is mathematically interpreted as a better agreement between DPLI and the Gaussian distribution.

### Outcome measures

Foot pain was measured by the well-validated self-reported Foot Health Status Questionnaire (FHSQ)[18,19] at baseline and after 3 months. The FHSQ is an accurate and reliable measure of foot-specific, health-related, quality-of-life scoring from 0 (worst score) to 100 points. The FHSQ also assesses footwear suitability and self-perception of general foot health. Scores of 85 and above on any item are considered to fall within normal ranges [18]. The change in pain score between baseline and 3 months was considered the primary outcome of this study.

### Data analysis

A multivariable general linear model (MANCOVA) was used for between group comparison by controlling the effect of participants’ characteristics (age, BMI, gender) and participants’ foot biomechanics (Foot Posture Index, DPLI, peak pressure, PTI, and the magnitude of 2nd peak pressure and the relative location of 2nd peak pressure as a percentage of the stance phase). In addition, ANCOVA was used for between group comparisons with adjustment by age. A multiple linear regression model (backward) was used to assess significant predictors to pain relief. The type of foot orthoses (custom-made foot orthoses =1 and Sham = 0) was inserted as the selection variable for the model and the custom orthoses group was selected for the final fitting. The dependent variable was the change in foot pain FHSQ score after 3 months wearing custom orthoses, compared to baseline. Independent variables included custom-orthoses participants’ characteristics (age and BMI), initial foot biomechanical parameters (Foot Posture Index and lunge test), and changes in plantar pressure after wearing orthoses (change in DPLI, maximum peak pressure, PTI, the magnitude of 2nd peak pressure and the relative location of 2nd peak pressure as a percentage of the stance phase), and initial foot pain score. To avoid artificially doubling the sample, a randomly selected right or left foot from each participant was used in final analysis. Since, the association between changes in pressure time-series and changes in pain was the main focus of this study, the constant parameter of the model was assumed to be zero. In other words, if the change in plantar pressure parameters was zero, we anticipated to have zero change in pain score in the follow-up assessment. Statistical analyses were performed using SPSS® version 20.

## Results

### Participants’ characteristics

Originally, 154 eligible participants were recruited. However, two participants were excluded from the final data analysis due to technical problems with the Pedar® system. Seventy nine from the control group (61% Female, Age = 49.5 ± 14.4 years, BMI = 27.4 ± 6.0 kg/m^2^) and 73 from the intervention group (52% Female, Age = 49.8 ± 14.4 years, BMI = 27.9 ± 5.8 kg/m^2^) were included in final analysis. At baseline no between groups significant difference was observed for any of the measures, suggesting effective randomization.

### Effect of foot orthoses on plantar pressure during walking

The effect of custom orthoses on change in plantar pressure time-series was demonstrated in our previous publication [8]. In summary, our preliminary results suggested that custom-made foot orthoses significantly increases DPLI on average by 44% compared to baseline (with shoes and no orthoses), while no improvement was observed in the control group. Both the control and treatment groups showed a decrease in the magnitude of peak pressure compared to baseline although the magnitude of reduction was higher in the treatment group than in the control group (8%; p < 0.001). The timing of the 2nd peak pressure was significantly later in the treatment group compared to baseline as well as compared to control (1.1%; p < 0.001).

### Effect of custom-made foot orthoses on foot pain relief

No significant difference was observed between groups for foot pain score at baseline (p = 0.098). Foot pain scores improved after three months follow-up for both groups (Table 1). However, the improvement of foot pain was, on average, 55% higher in the treatment group compared to the control group (p = 0.005, mean difference = 11.1, 95% CI = 3.3 to 19.0). No correlation between change in pressure magnitude and change in symptoms was found in either group (r < 0.1, p = 0.494). On the same note, no correlation was found between change in PTI and change in pain (r < 0.1, p = 0.412). However, redistribution of plantar pressure, measured with the DPLI, had a significant effect on pain relief (r = 0.28, p = 0.001).

**Table 1:** Difference between control and intervention groups at baseline and 3-months follow-up

	**Control group N = 79**	**Intervention group N = 73**	**Mean difference (p-value)**	**95% CI**
	**Mean ± SD**	**Mean ± SD**		
**Gender (% Female)**	60.8%	52.1%	8.7(0.327)	-
**Age (years)**	49.5 **±** 14.4	50.1 **±** 14.0	0.22(0.923)	(−4.3,4.8)
**BMI (kg/m2)**	27.4 **±** 6.0	27.9 **±** 5.8	0.5(0.583)	(−1.3,2.4)
**Initial pain score**	46.7 **±** 18.1	41.9 **±** 17.7	−4.8(0.122)	(−10.8,1.3)
**Follow-up pain score**	67.0 **±** 22.2	73.4 **±** 24.9	6.4(0.098)	(−1.2,13.9)
**Change in pain**	20.3 **±** 22.7	31.5 **±** 26.1	11.1(0.006)	(3.3,19.0)
**Results after adjusting by age**				
**Initial pain score**	45.3	41.1	−4.1(0.180)	(−10.2,1.9)
**Follow-up pain score**	65.3	73	7.4 (0.061)	(−.3,15.0)
**Change in pain**	20.0	31.5	11.5(0.005)	(3.5,19.5)

### Prediction of foot pain relief with custom-made foot orthoses

**Figure 3:**
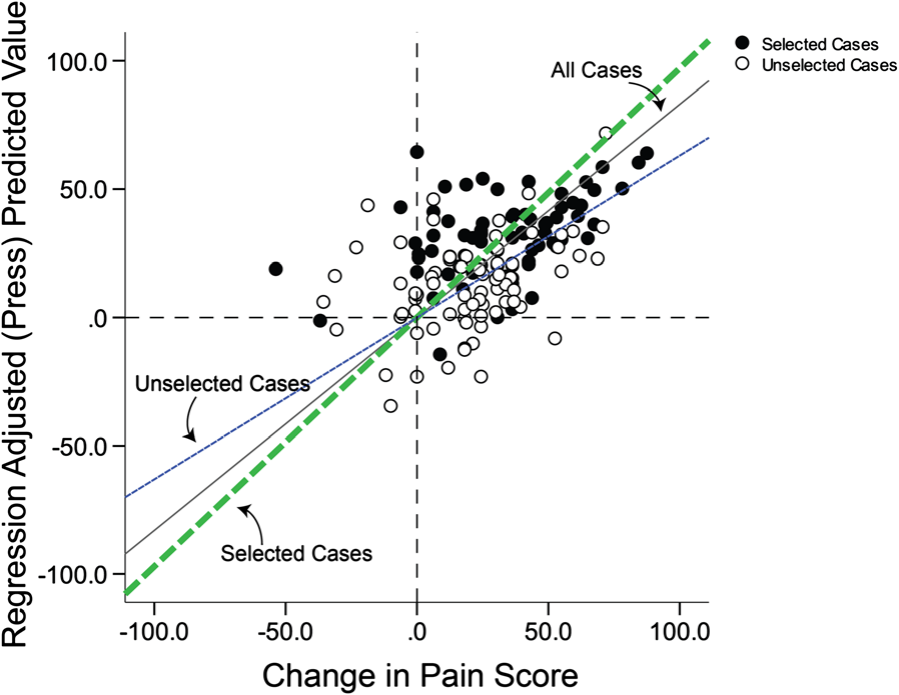
**The model output for prediction of foot pain score change post custom orthoses intervention.** Selected cases (filled circles) represent the CFO group and unselected cases (empty circles) represent the control group. The diagonal dash-lines represent the best linear fit for each selected and unselected cases. The solid diagonal line represents the best linear fit for both groups altogether.

Table 2 summarizes the significant predictors of pain reduction in the intervention group using the multiple linear regression model described in the method. Results suggest that change in foot pain with custom orthoses could be modeled with relatively good accuracy (R-square = 0.726, standard error of the estimate = 22.79) by initial foot pain FHSQ score, BMI, Foot Posture Index, and changes in DPLI and PTI (p < 0.05). Changes in peak pressure magnitude and the location and magnitude of the 2nd peak as well as baseline values of ankle range (lunge test) and age were not significant predictors. Figure 3 illustrates the scatter plot of association between foot pain score change and the independent variables described above.

**Table 2:** Significant predictors of pain relief with custom-made foot orthoses

**Coefficients**^**a,b,c**^										
**Model**	**Unstandardized coefficients**		**Standardized coefficients**	**t**	**Sig.**	**95.0% confidence interval for B**		**Correlations**		
	**B**	**Std. error**	**Beta**			**Lower bound**	**Upper bound**	**Zero-order**	**Partial**	**Part**
BMI	1.379	.269	.942	5.119	.000	.841	1.917	.782	.539	.335
Dynamic Plantar Loading Index Change	46.868	18.072	.253	2.593	.012	10.766	82.970	.667	.308	.170
PTI Change	−3.038	.987	−.389	−3.077	.003	−5.010	−1.066	−.713	−.359	−.201
Foot Posture Index	2.008	1.018	.251	1.972	.053	−.026	4.042	−.584	.239	.129
FHSQ initial foot pain score	−.475	.139	−.514	−3.424	.001	−.752	−.198	.602	−.394	−.224

a. Dependent Variable: Change in pain score.

b. Linear Regression through the Origin.

c. Selecting only cases for which footwear_type = 1 (CFO).

**Figure 4:**
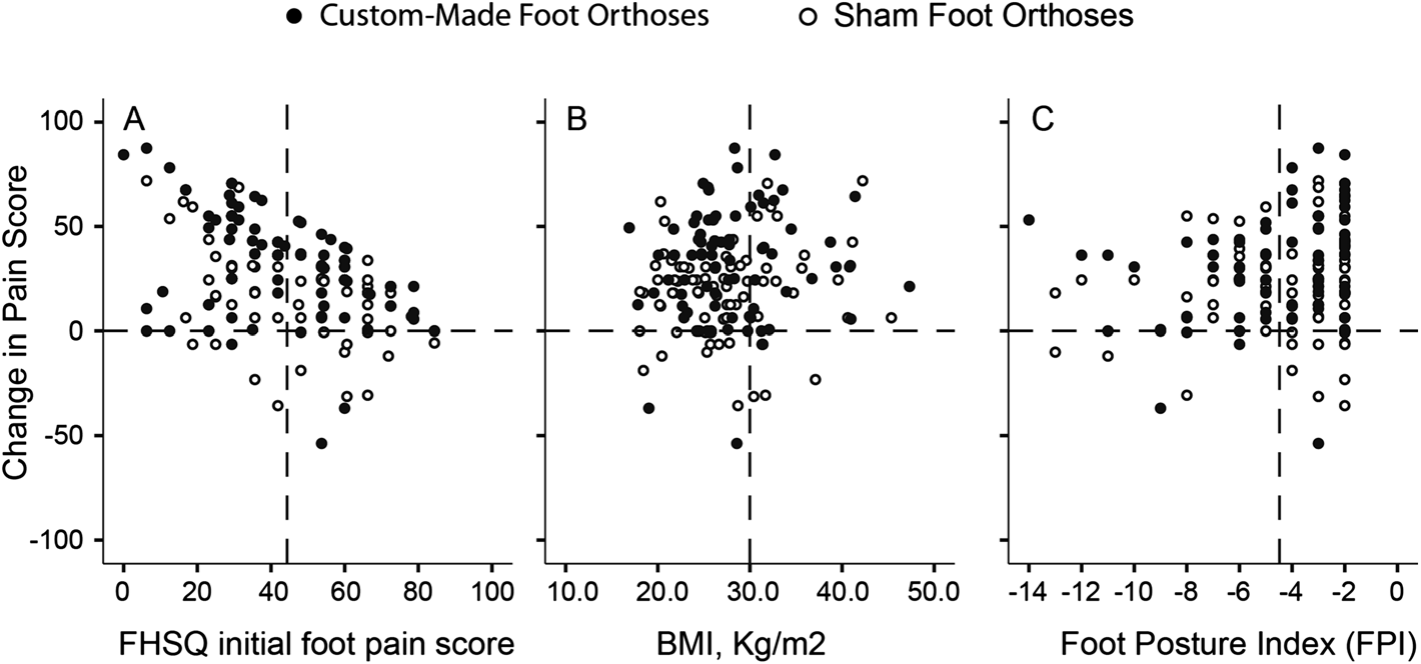
**The association between foot pain score change post custom orthoses intervention as a function of (A) FHSQ initial foot pain score, (B) BMI, and (C) Foot Posture Index.** The vertical dash line represents the mean value.

Figure 4 illustrates the scatter plot for the association between change in pain and baseline parameters, including participants’ initial pain score (Figure 4A), BMI (Figure 4B), and Foot Posture Index (Figure 4C). Results suggest that effective pain reduction depends on initial pain score. In other words, those who have higher pain levels may benefit more from the prescribed orthoses (R-partial = −0.4). On the same note, pain relief was mediated by an increase in BMI and lower Foot Posture Index (i.e., worse pes cavus foot deformity) after intervention with prescribed custom-made foot orthoses (R-partial = 0.54 for BMI and 0.24 for Foot Posture Index).

**Figure 5:**
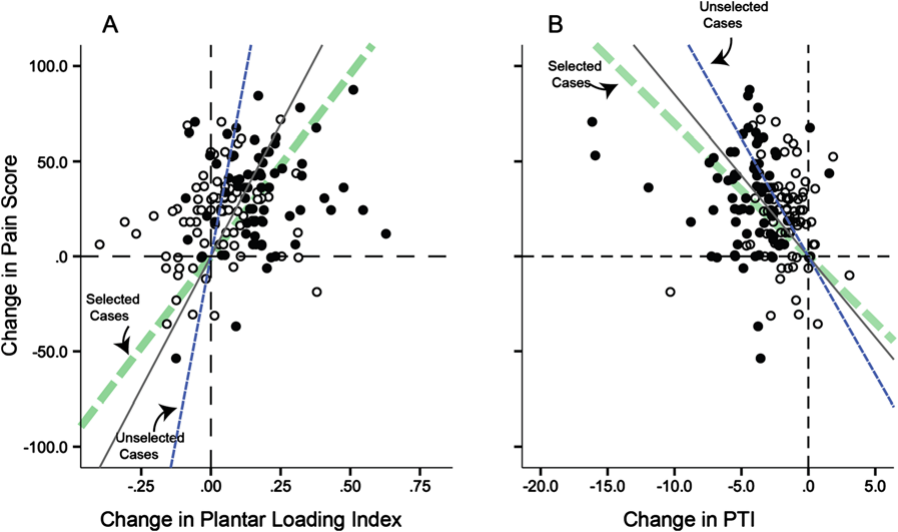
**The association between foot pain score change post custom orthoses intervention as a function of (A) change in Dynamic Plantar Loading Index and (B) change in pressure time integral (PTI).** The diagonal dash-lines represent the best linear fit for each selected and unselected cases. The solid diagonal line represents the best linear fit for both groups altogether.

Figure 5 illustrates the associations between change in pain and change in DPLI (Figure 5A) and PTI (Figure 5B). Results suggest that pain in the intervention group was reduced by increasing the DPLI (B = 46.8(18,1), p = 0.012, R-partial = 0.31, Zero-order R = 0.67). On the same note, pain may be reduced by reducing PTI (B = −3.0(0.99), p = 0.003, R-partial = −.359, Zero-order R = −0.71).

## Discussion

In this secondary data analysis of a randomized clinical trial of custom-made foot orthoses for treatment of painful pes cavus, we investigated possible predictor variables for treatment response in the custom-made foot orthoses group. We aimed to describe both demographic and biomechanical mediators of pain-relief afforded with custom-made foot orthoses usage. Our final model described 73% (R^2^) of the variance in pain relief with custom-made foot orthoses and consisted of higher initial pain level, higher BMI, cavoid foot alignment as measured by Foot Posture Index, and changes in both the DPLI (e.g. increased towards healthy participants’ value) and decreased PTI. As far as we are aware, we are the first group to describe as much variance in pain relief with custom-made foot orthoses.

In our original trial [5], we postulated that custom-made foot orthoses mediated pain relief via reduced plantar pressures. Our subsequent analysis of 66 people with idiopathic cavus foot deformity that were custom-made foot orthoses responders did not find an association between pressure variables and pain relief [7]. However, this analysis as well as other studies [20] may have been underpowered. Postema and colleagues studied 42 participants with metatarsalgia and examined their pain relief and plantar pressures in response to various shoe and foot orthoses conditions. In a subset of 18 patients that reported pain, they did not detect significant correlations although one value approached significance (p = 0.06) [20]. Furthermore our prior definition of ‘responders’ required a change of 10 points in pain and function (n = 44) or final pain score greater than 85 (n = 20) [7].

Alternatively, Jannink and colleagues studied 77 patients with degenerative foot conditions treated with custom-made orthopaedic shoes and measured their pain relief and plantar pressures after 3 months use. They found 27% of the variance in walking pain could be attributed to average pressure [21]. While they also detected a significant decrease in stance time, this translated into a less than 10% difference in walking speed and was not believed to be clinically significant [21]. We also found differences in stance time indicating a faster walking speed following custom-made foot orthoses usage, and adjustments for this difference did not change our final model.

DPLI and PTI were the only biomechanical variables retained in the final model explaining pain relief with custom orthoses. We believe these results provide mechanistic support for custom-made foot orthoses in reducing pain in patients with pes cavus. Mechanistic theories for pain-relief from custom-made foot orthoses include resisting or facilitating motion; plantar pressure reduction; altered muscle activity; and enhanced proprioception [2]. Nigg further developed a theoretical framework for injuries associated from foot pronation for impact and movement control. Impact forces produce muscle tuning reaction prior to impact and muscle activation during contact provide a preferred joint movement path [22]. Our previous kinematic studies in healthy participants support these theoretical constructs as we found foot orthoses reduced gait initiation distance and time [23].

We also found that custom-made foot orthoses usage increased self-selected walking speeds, reduced inter-cycle walking speed variability, and reduced center-of-mass oscillation in the medial and lateral direction [3]. Our subsequent kinetic studies supported these concepts in painful pes cavus patients with the DPLI redistributing pressure [8]. In the present study we found both PTI and DPLI predicted pain relief from custom-made foot orthoses reflecting both the quantity and shape of pressure redistribution. In our prior studies, self-selected gait speed increased in healthy and in patients with pes cavus following foot orthoses usage [3,8,23]. In the present study, after adjusting for stance time changes, PTI and DPLI were still retained. In this case, PTI may reflect the temporal redistribution of pressures over time. Our prior studies also suggested that the DPLI was unchanged with changes in gait speed [8,9]. Thus, DPLI appears to reflect a person’s plantar pressure probability distribution to a Gaussian distribution as its effects were independent of PTI. This also supports Nigg’s theoretical framework of a preferred joint movement path or shape of pressure redistribution.

There are limitations to our study. This is a secondary analysis of a randomized controlled trial. While the trial was not initially designed or powered to detect these *post-hoc* questions, the design offers strengths over previously underpowered studies investigating the mechanism of custom foot orthoses on pain relief after orthotic therapy [7,20].

## Conclusion

In this study we have identified a range of demographic and biomechanical predictors of pain-relief with the use of custom-made foot orthoses in people with painful cavus foot deformity. Our final model described 73% (R^2^) of the variance in pain relief from custom-made foot orthoses and consisted of initial pain level, BMI, foot alignment, and changes in both the Dynamic Plantar Loading Index and PTI. Our findings add to the growing body of literature providing mechanistic support for the effect of custom orthoses on a variety of painful foot conditions.

## Abbreviations

CFO: Custom-made foot orthoses; DPLI: Dynamic plantar loading index; BMI: Body Mass Index; FPI: Foot Posture Index; PTI: Pressure time integral; FHSQ: Foot Health Status Questionnaire; CI: Confidential interval.

## Competing interests

The authors declare that they have no competing interests.

## Authors’ contributions

JB conducted the main study and shared the data for the purpose of this study. He also contributed in the exploration of data, writing the manuscript, and discussion. BN explored and analyzed the data and wrote the manuscript. He developed the main algorithm used to assess the dynamic planar loading behavior during walking. JW contributed in exploring the data and writing the manuscript and discussion. All authors read and approved the final manuscript.
